# A Gallbladder Volvulus Presenting as Acute Cholecystitis in a Young Woman

**DOI:** 10.7759/cureus.9435

**Published:** 2020-07-28

**Authors:** Pham Hong Duc, Pham Hong Van, Huynh Quang Huy

**Affiliations:** 1 Radiology, Hanoi Medical University, Hanoi, VNM; 2 General Planning, National Hospital of Traditional Medicine, Hanoi, VNM; 3 Radiology, Pham Ngoc Thach University of Medicine, Ho Chi Minh City, VNM

**Keywords:** gallbladder volvulus, abdomen pain, cholecystitis, appendicitis, fitz-hugh-curtis syndrome

## Abstract

Gallbladder volvulus is rare and known to occur when there is a rotation of the gallbladder along the axis of the cystic vascular pedicle. Except for isolated cases reported in childhood, this disease is more frequently encountered in the elderly, especially in women. Therefore, most cases in the literature describe the clinical and radiographic features of gallbladder volvulus in this age group. In this subject, manifestations of abdominal pain are variable. Typically, right upper quadrant pain leads towards the diagnosis of cholecystitis, and diagnosis may be performed on imaging such as computed tomography (CT) scan with multi-plane CT reconstruction. However, preoperative diagnosis of gallbladder torsion remains difficult, and in most cases, especially in young women where it is rare, the clinical and radiographic features seem to differ from gallbladder torsion in the elderly. We report a case of gallbladder volvulus in a 26-year-old woman and review the clinical and radiological aspects of this disease.

## Introduction

Gallbladder volvulus is a very rare clinical condition of the hepatobiliary system. The most commonly accepted mechanisms for this disease include variant anatomy of the long mesentery gallbladder. In this case, it attaches only at the cystic duct and its associated vascular cystic pedicle, allowing for it to rotate along its axis; this is known as a floating or pedunculated gallbladder [1]. This predisposition is not just a congenital explanation for possible occurrence in children; loss of fatty and elastic tissue and liver atrophy with or without increased peristalsis from the surrounding bowel and kyphoscoliosis are factors that help explain gallbladder volvulus in older women being more common [1-3]. Indeed, Reilly et al. reviewed a total of 324 patients related to gallbladder torsion reported since 1898. The median age at presentation is 77 years (range: five days to 100 years). The sample is more common in adult patients accounting for 84% (aged > 18 years) and more frequently in women than in men (4:1); 16% occurred in pediatric patients (aged 0-18 years) and was more common in boys than girls (2.5:1) [4]. We note that the first gallbladder volvulus described as a floating gallbladder was reported in 1898 by Wendel in a 23-year-old pregnant woman [5].

Although torsion of the gallbladder is a very rare occurrence in young women, we report a 26-year-old woman in whom we confirmed a post-operative diagnosis of gallbladder volvulus. It initially presented as acute appendicitis and a Fitz-Hugh-Curtis syndrome, followed by the findings of acute cholecystitis on imaging. Also, in this case, we would like to contribute enriching the bank of these rare cases and providing a part of the collection of very useful signs that radiologists, as well as surgeons, can memorize in mind in case of differences.

## Case presentation

A 26-year-old unmarried woman with a history of treated salpingitis eight years ago presented to the emergency department with several hours of sudden onset abdominal pain. This pain was described as sharp and intermittent, localized to the right side of the abdomen, without vomiting or fever. On physical examination, her vital signs were stable, the abdomen was not distended, with mild tenderness of involuntary muscular resistance in the right iliac fossa. The urgent abdominal ultrasonography (US), including the appendix, gallbladder, and pelvic gynecology, were unremarkable. The patient presented vaginal discharge, no missed period, on amenorrhea, and a negative quick stick test. Laboratory studies showed an inflammatory condition with leukocytes of 11 g/L , neutrophils accounting for 87%, and C-reactive protein of 167 mg/L. Other tests, including hepatic and pancreatic enzymes, were within the normal range. The patient was monitored due to atypical appendicitis with intravenous fluid resuscitation.

**Figure 1:**
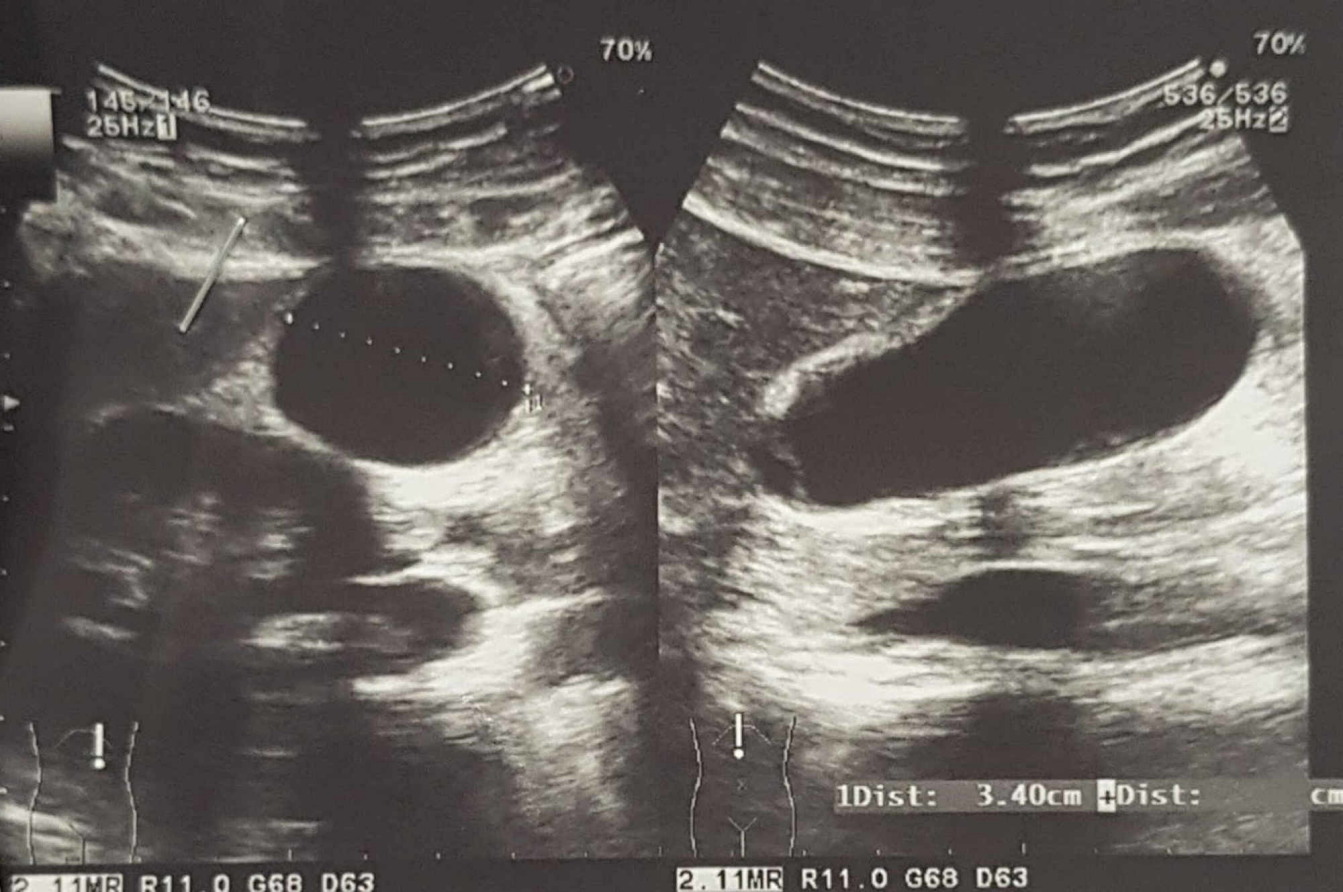
Abdominal ultrasonography The ultrasonography revealed an abnormally enlarged gallbladder without gallstones and a slightly diffuse thickened gallbladder wall

After about the sixth hours of admission, because the patient complained of more pain, including the right upper quadrant and mild hyperthermia, an abdominal ultrasound (US) was performed. This demonstrated no signs of appendicitis but showed a markedly distended gallbladder without gallstones or sludge and slight wall thickening with non-specific pericholecystic fluid that was likely reactive (Figure 1). The US was followed by atypic acalculous cholecystitis with intravenous antibiotics in keeping with the hospital protocol for the treatment of intra-abdominal sepsis.

**Figure 2:**
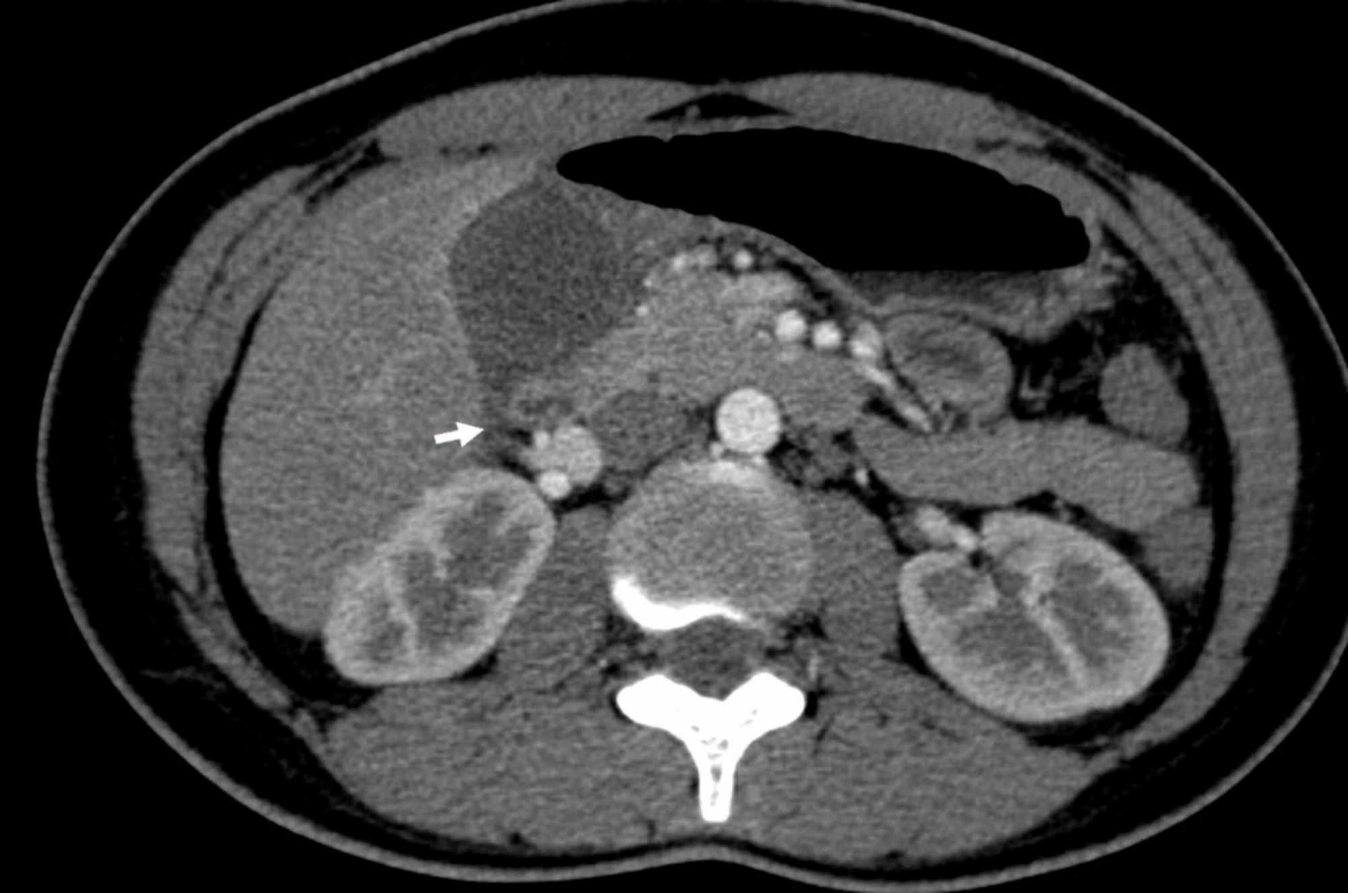
Abdominal enhanced CT The enhanced CT demonstrated a distended gallbladder measuring 82 x 42 mm, and a non-enhanced wall associated with a pericholecystic effusion (arrow). Abbreviation: CT, computed tomography

After about 15 hours of admission, the patient had relief from the pain, fever, and abdominal distention. Thus, an abdominal computed tomography (CT) scan was recommended, which revealed a distended gallbladder measuring 82 x 42 mm, a normal fossa with a thickened non-enhancing wall, the presence of pericholecystic effusion, and fluid in the Douglas pouch (Figure 2). The CT results and clinical manifestation led us to suspect Fitz-Hugh-Curtis syndrome, so patient monitoring was continued.

**Figure 3:**
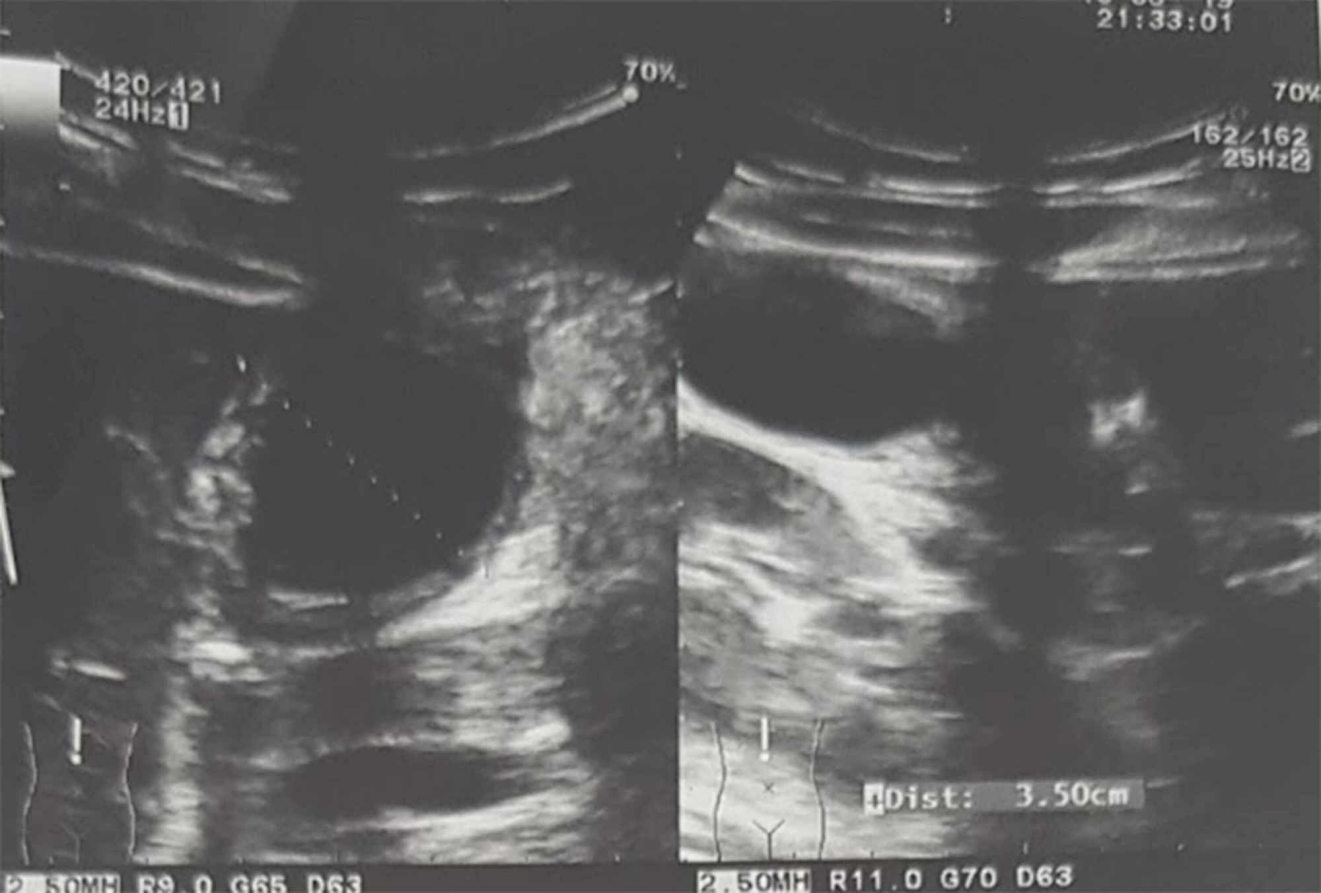
Sequential abdominal ultrasonography The ultrasonography showed a more marked distended gallbladder and thickened gallbladder wall

After about 27 hours of admission, because the abdominal pain was not improved, the second abdominal US was ordered. This showed a more distended gallbladder with a thickened wall (5 mm) and pericholecystic fluid with fluid in the right iliac fossa and the Douglas pouch (Figure 3). However, there were no signs of appendicitis. These results suggested acute cholecystitis. As this was not confirmed by CT, monitoring of her was continued, delaying her operation.

**Figure 4:**
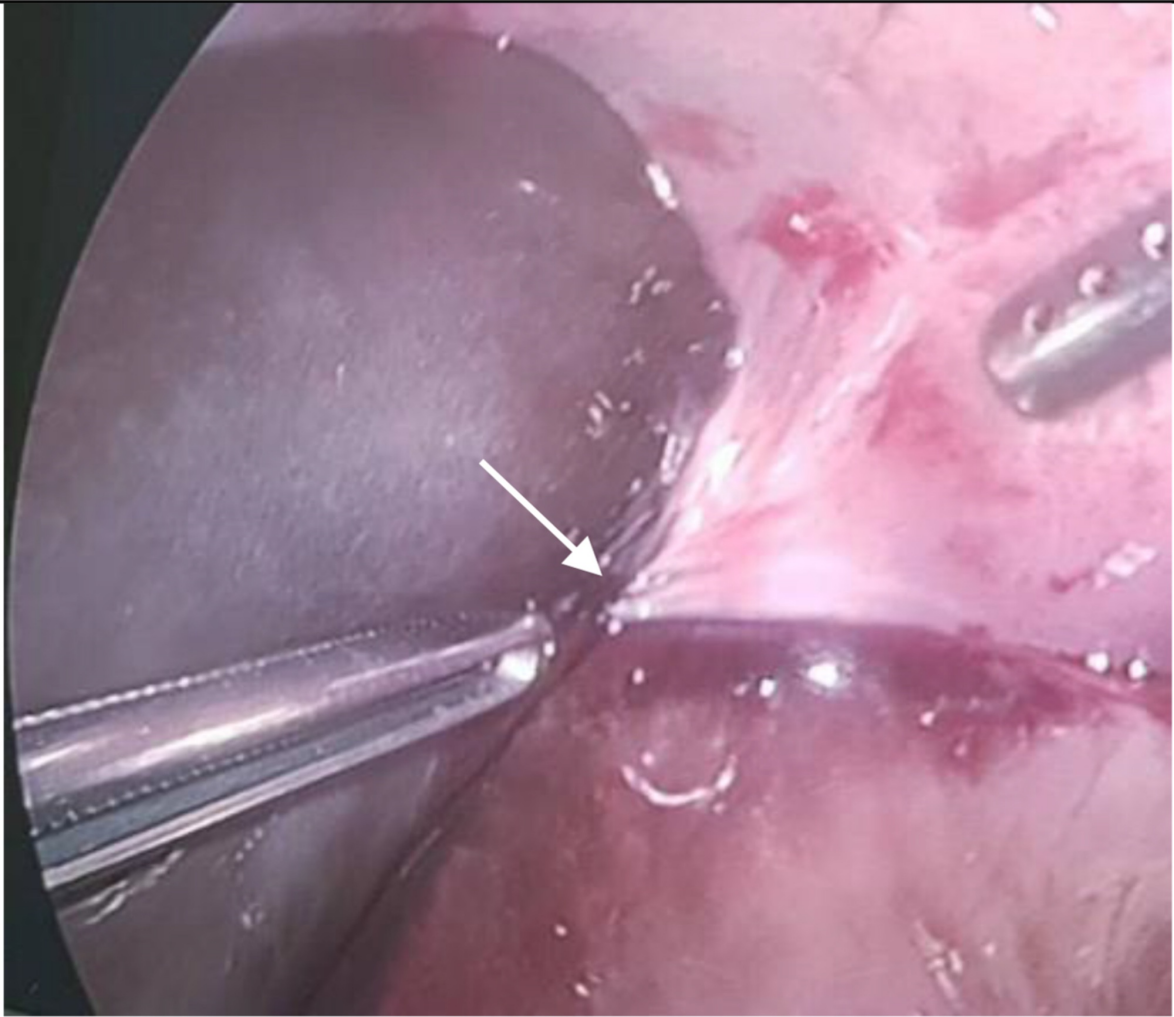
Intra-operative images showing distended and gangrenous avascular gallbladder wall with clockwise torsion 360 degrees around of the cystic vascular pedicle (arrow)

**Figure 5:**
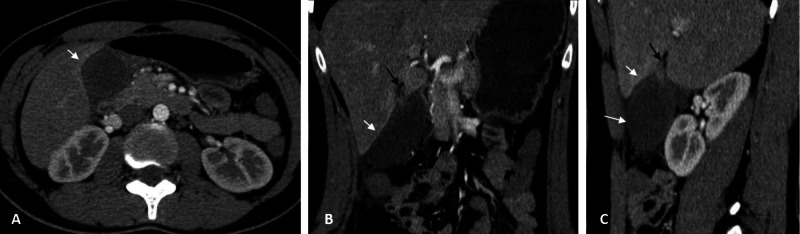
Gallbladder CT A review of the gallbladder CT in the arterial phase, showing axial (A), coronal (B), and sagittal (C) views with a beak sign at the cystic pedicle of the gallbladder (black arrow), slightly floating gallbladder (long white arrow) without horizontal laying of the gallbladder. Note the enhanced rim of the liver capsule (short white arrow) separating from the non-enhanced gallbladder wall is a thin layer of fluid. Abbreviation: CT, computed tomography

Finally, the patient was operated at the 42nd hour after admission, in light after observing the clinical symptoms, including positive Murphy’s sign of typical acute cholecystitis. Surgical laparoscopy showed the distended gangrenous gallbladder caused by a tightly twisted clockwise direction of 360 degrees on the cystic vascular pedicle, leaving the gallbladder body suspended freely from the liver bed with a long mesenteric attachment (Figure 4). In addition, the gallbladder had not perforated, and the presence of several pseudomembranous tissues in the peritoneal cavity around the gallbladder and a small amount of peritoneal fluid in the pouch of Douglas was noted. The hepatic capsule did not show inflammation. The appendix and salpinx appeared normal. Reviewing a multi-plane CT reconstruction (Figure 5), we found a tiny beak appearance at the cystic vessels. In addition, we noticed an enhanced rim of the hepatic capsule, which may be due to an inflammatory reaction, that was sharply contrasting with the non-enhanced gallbladder wall and a thin layer of fluid. This suggested a gallbladder body free from the bed liver. A cholecystectomy was performed, and drainage was placed in the right subhepatic space. The postoperative course was uneventful. Histology revealed transmural gallbladder necrosis without evidence of lithiasis. She was discharged on postoperative day five without complications.

## Discussion

Gallbladder volvulus patients frequently present to the emergency department with acute abdominal pain. Typically, the symptoms are the sudden onset of severe upper-right quadrant pain. However, although rare, the clinical findings may also appear similar to acute appendicitis, such as that reported, presenting right lower quadrant pain with a palpable mass in an elderly woman that may cause a wrong determination of the location of the distended gallbladder in the right iliac fossa [6]. In our case, from the initial presentation of the patient, such as inflammatory markers and signs of localized pain in the right lower quadrant abdomen suggesting acute appendicitis. Another clinical diagnosis was also mentioned in a woman of childbearing age with vaginal discharge who presented with acute pain in the upper right abdomen; a diagnosis of Fitz-Hugh-Curtis syndrome is not rare, occurring for 64% of patients where a first diagnostic impression of acute cholecystitis is made (46%) [7].

The preoperative diagnosis of gallbladder volvulus accounts for 21% to 27% of cases but remains difficult because the nonspecific clinic and radiographic findings may be similar to those of acute infective pathology [3,4,8]. Most of the preoperative diagnoses are for acute cholecystitis (67%), or less common conditions such as acute appendicitis (6%) and bowel obstruction (6%) [8]. Although without lithiasis, accounting for 68% to 78% cases of gallbladder volvulus cases, which is considered one of the factors suggesting diagnosis, it is still difficult to diagnose and often leads to a diagnosis of acute acalculous cholecystitis as gallbladder torsion is rare [3,4]. This is especially true due to limited experience for radiologists on radiographic findings.

In most of the cases, the preoperative diagnosis of gallbladder volvulus was based on radiographic findings such as US, CT, and/or magnetic resonance imaging (MRI). US is the preferred modality due to its convenience. It is possible to monitor the progression of acute cholecystitis, but it might not always be possible to distinguish from gallbladder torsion as both might show distended gallbladder and thickened gallbladder wall as in our case. MRI also helps to add diagnostic signs such as an invagination-like image identified the neck of the gallbladder or a v-shaped distortion of the extrahepatic bile ducts and is therefore considered as the diagnostic method of choice in a pregnant patient [9,10].

An abdominal CT scan with multi-plane CT reconstruction is a strong indicator of the diagnosis of a gallbladder volvulus. Most of the cases are described in older women with the following criteria: distended gallbladder, floating or pedunculated gallbladder, a beak or whirlpool signs at the cystic pedicle, low and horizontal laying of the gallbladder, thickened and non-enhanced gallbladder wall, and an absence of gallstones [1,2,11-13]. In one rare case, they reported gallbladder torsion diagnosed with sequential abdominal CT showing cystic duct rotated of approximately 180 degrees from the left to the right side of the gallbladder [14].

In our case, as well as in some cases in the literature, gallbladder torsion is extremely rare in young women and often did not see the radiographic features suggestive of gallbladder torsion as in the elderly. It is often only diagnosed after surgery when retrospective images can be identified [4,10,15].

## Conclusions

Clinical manifestations may be confounding factors, and atypical radiographic findings also delay the diagnosis of this disease. Through our case, we have shown that careful image analysis is important to the correct diagnosis. Therefore, although gallbladder volvulus is very uncommon, its diagnosis should be considered in all patients presenting with right side abdominal pain, especially in young women, which could be misdiagnosed with acute acalculous cholecystitis.
